# Translation of the updated clinical frailty scale 2.0 into Danish and implications for cross-sectoral reliability

**DOI:** 10.1186/s12877-021-02222-w

**Published:** 2021-04-21

**Authors:** Anders Fournaise, Søren Kabell Nissen, Jørgen T. Lauridsen, Jesper Ryg, Christian H. Nickel, Claire Gudex, Mikkel Brabrand, Lone Musaeus Poulsen, Karen Andersen-Ranberg

**Affiliations:** 1 Department of Cross-sectoral Collaboration, Region of Southern Denmark, 7100 Vejle, Denmark; 2 Department of Geriatric Medicine, Odense University Hospital, 5000 Odense, Denmark; 3 Epidemiology, Biostatistics and Biodemography, Department of Public Health, University of Southern Denmark, 5000 Odense, Denmark; 4 Institute of Regional Health Research, Centre South West Jutland, University of Southern Denmark, 6700 Esbjerg, Denmark; 5 Department of Emergency Medicine, Hospital of South West Jutland, 6700 Esbjerg, Denmark; 6 Department of Business and Economics, University of Southern Denmark, 5230 Odense, Denmark; 7 Department of Clinical Research, University of Southern Denmark, 5000 Odense, Denmark; 8 Emergency Department, University Hospital Basel, University of Basel, 4031 Basel, Switzerland; 9 Open Patient data Explorative Network (OPEN), Region of Southern Denmark, 5000 Odense, Denmark; 10 Department of Emergency Medicine, Odense University Hospital, 5000 Odense, Denmark; 11 Department of Anaesthesiology, Zealand University Hospital, 4600 Køge, Denmark; 12 Collaboration for Research in Intensive Care (CRIC), 2100 Copenhagen, Denmark

**Keywords:** Cross-sectoral collaboration, Continuity of care, ISPOR translation, Validation, Reliability, Geriatrics, Clinical frailty scale

## Abstract

The Clinical Frailty Scale, which provides a common language about frailty, was recently updated to version 2.0 to cater for its increased use in areas of medicine usually involved in the care and treatment of older patients. We have previously translated the Clinical Frailty Scale 1.2 into Danish and found inter-rater-reliability to be excellent for primary care physicians, community nurses, and hospital doctors often involved in cross-sectoral collaborations. In this correspondence we present the Danish translation and cultural adaption of the Clinical Frailty Scale 2.0. Our recent findings on cross-sectoral inter-rater reliability for the Clinical Frailty Scale 1.2 are likely also applicable for the Clinical Frailty Scale 2.0.

**Supplementary Information:**

The online version contains supplementary material available at 10.1186/s12877-021-02222-w.

## Background

The previous version of the Clinical Frailty Scale (CFS 1.2) was recently translated into Danish and published alongside results from a cross-sectoral inter-rater reliability study among primary care physicians, community nurses, and hospital doctors [1]. We found excellent inter-rater reliability across these four groups of health care professionals, supporting the notion that the CFS has the potential to serve as a common reference tool when treating and rehabilitating older patients. Since then, the developers of the CFS have modified the instrument (CFS 2.0) to facilitate its use as a triage tool regarding intensive care treatment for older patients with COVID-19 [2, 3] and to increase its relevance in areas of medicine not usually involved in the assessment of frailty [4, 5]. Here, we present a translation of the CFS 2.0, briefly summarize changes between the two versions, and discuss possible implications for the cross-sectoral reliability.

## Results

**Fig. 1:**
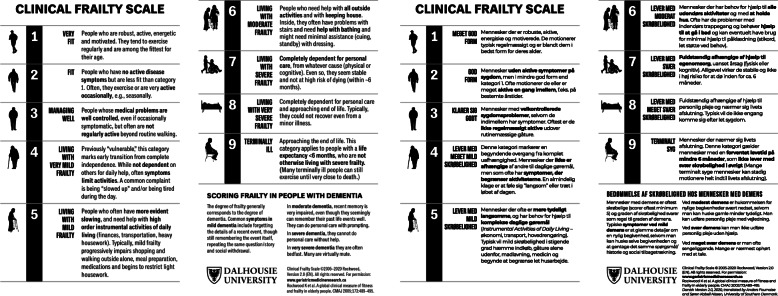
Legend: The Clinical Frailty Scale 2.0 source instrument in English (left) and the Danish translation (right). Printed with permission from copyright holder

As with the CFS 1.2, we translated the CFS 2.0 in accordance with the ISPOR guidelines [6]. This process is designed to ensure cultural and conceptual compliance with the source instrument [7, 8]. The CFS 2.0 and its Danish translation (CFS-DK 2.0) are presented in Fig. 1, and the Danish translation report is available in the ‘Additional file 1’. In ‘Additional file 2’, the differences between CFS 1.2 and CFS 2.0 are highlighted in the English and Danish versions.

## Discussion

The CFS is validated for assessing the *habitual* health state of patients rather than the state of acute illness [7], a caveat not immediately evident from the 9-level pictogram scale. To remedy this, the developers have revised the level headings in CFS 2.0 to indicate that the health care professional should consider the patient’s baseline health state (for example, “vulnerable” has become “living with very mild frailty”) [3]. However, health care professionals will still be faced with a challenge to determine when the health state of the patient changed from habitual to acutely ill. This tipping point varies from patient to patient, and for the CFS 2.0 it remains relevant for clinicians to include information from relatives and from other health care professionals in cross-sectoral collaborations.

Other changes include additional information on differentiating between severe and very severe dementia (for health care professionals who are less familiar with the spectrum of dementia diseases) and the writing out of “instrumental activities of daily life”, which was previously presented just in its abbreviated form, “IADL”). From our experiences of translating both the CFS 1.2 and 2.0, we agree with instrument developers that the CFS 2.0 is likely to be more relevant than CFS 1.2 for assessing acutely ill older patients and patients living with severe dementia [3].

In our inter-rater reliability study on CFS-DK 1.2 we found little variance among raters when rating CFS levels 4 and 5, the threshold at which the term “frail” is included in headings [1]. However, a recent study on older (≥ 80 years of age) patients in intensive care units found high variance among raters when differentiating these two levels using the CFS version 1.2 [9]. Though the study also confirms a high ICC for the CFS 1.2 in the intensive care setting, differentiating CFS level 4 and 5 may pose a challenge in the CFS 2.0 despite the change of heading in CFS level 4 from “vulnerable” to “living with very mild frailty” [3].

We consider the differences between CFS 1.2 and 2.0 versions to be minor and that the results of our recent inter-rater reliability study on CFS-DK 1.2 are likely still applicable to the 2.0 version [1]. The inter-rater reliability study used clinical vignettes to describe individuals in their habitual state, and raters were introduced to the CFS 1.2 (including an explanation of the abbreviation IADL and the importance of scoring the subject according to habitual health state) before they rated the vignettes. Furthermore, none of the vignettes described severely or very severely demented patients (which the CFS 2.0 now distinguishes).

## Conclusion

A Danish translation of the updated Clinical Frailty Scale 2.0 is now available, and we refer potential users to our recent cross-sectoral inter-rater reliability study performed with the CFS 1.2. We believe the results of this study will still be relevant for the CFS-DK 2.0 in view of the minor differences between the versions.

## Supplementary Information

**Additional file 1.** Final report - ISPOR translation of Clinical Frailty Scale 2.0 into the Danish language.

**Additional file 2.** Clinical Frailty Scale version 1.2 and 2.0 in English and Danish, with the differences between the 1.2 and 2.0 versions highlighted.

## Data Availability

The datasets used and/or analysed during the current study are available from the corresponding author on reasonable request.

## Abbreviations

CFS: Clinical Frailty Scale
IADL: Instrumental Activities of Daily Life
ICC: Intraclass Correlation Coefficient
