# Supplementary material for: Translation of the updated clinical frailty scale 2.0 into Danish and implications for cross-sectoral reliability
Source: BMC Geriatr. 2021 Apr 21;21:269. doi: 10.1186/s12877-021-02222-w (PMC8059120; doi:10.1186/s12877-021-02222-w)
Supplement: Supplementary file 1 — Additional file 1. Final report - ISPOR translation of Clinical Frailty Scale 2.0 into the Danish language. [file 12877_2021_2222_MOESM1_ESM.pdf]

Translation of the updated Clinical Frailty Scale 2.0 into Danish and implications for cross-sectoral reliability

Anders Fournaise (ORCID - 0000-0002-4754-7500)* [1,2,3], Søren Kabell Nissen (ORCID - 0000-0003-3722-908X) * [4,5], Jørgen T. Lauridsen (ORCID - 0000-0001-9889-6236) [6], Jesper Ryg (ORCID - 0000-0002-8641-3062) [2,9], Christian H. Nickel (ORCID - 0000-0001-6619-9284) [4,8], Claire Gudex (ORCID - 0000-0003-3881-9890) [9,10], Mikkel Brabrand (ORCID - 0000-0002-3340-8251) [4,5,7,9], Lone Musaeus Poulsen (ORCID - 0000-0002-7030-3395) [11,12], Karen Andersen-Ranberg (ORCID - 0000-0003-1970-7076) [2,3,9]

[1] Department of Cross-sectoral Collaboration, Region of Southern Denmark, 7100 Vejle, Denmark

[2] Department of Geriatric Medicine, Odense University Hospital, 5000 Odense, Denmark

[3] Epidemiology, Biostatistics and Biodemography, Department of Public Health, University of Southern Denmark, 5000 Odense, Denmark

[4] Institute of Regional Health Research, Centre South West Jutland, University of Southern Denmark, 6700 Esbjerg, Denmark

[5] Department of Emergency Medicine, Hospital of South West Jutland, 6700 Esbjerg, Denmark

[6] Department of Business and Economics, University of Southern Denmark, 5230 Odense, Denmark

[7] Department of Emergency Medicine, Odense University Hospital, 5000 Odense, Denmark

[8] Emergency Department, University Hospital Basel, University of Basel, 4031 Basel, Switzerland

[9] Department of Clinical Research, University of Southern Denmark, 5000 Odense, Denmark

[10] Open Patient data Explorative Network (OPEN), Region of Southern Denmark, 5000 Odense, Denmark

[11] Department of Anaesthesiology, Zealand University Hospital, Koege, Denmark

[12] Collaboration for Research in Intensive Care (CRIC), Copenhagen, Denmark

*co-first authors

Corresponding author

Anders Fournaise, MSPH

Department of Cross-sectoral Collaboration, Region of Southern Denmark, Damhaven 12, 7100 Vejle, Denmark

E-mail: anders.fournaise@rsyd.dk / 0045 2482 4315

Contact information: Anders Fournaise – anders.fournaise@rsyd.dk / Søren Kabell Nissen – sknissen@health.sdu.dk

Additional file 1
Final report
Translation of Clinical Frailty Scale 2.0 into the Danish language

Introduction

In a recent issue of the BMC Geriatrics, we presented a translation of the Clinical Frailty Scale 1.2 (CFS) into Danish and tested the inter-rater-reliability for primary care physicians, community nurses and hospital doctors often involved in cross-sectoral collaborations. However, very recently the scale was updated to a version 2.0. Below we present the ISPOR final report for translating the CFS 2.0 into Danish.

The study group has recently tested the cross-sectoral reliability of the CFS 1.2, and because the CFS 2.0 only slightly differs from the CFS 1.2 no further reliability test has been performed - https://bmcgeriatr.biomedcentral.com/articles/10.1186/s12877-020-01850-y.

The translation of the CFS 2.0 were performed using the 10-step ISPOR Principles of Good Practice for the Translation and Cultural Adaptation Process for Patient-Reported Outcomes Measures (Wild et al. 2005).

ISPOR CFS group

Project manager: Anders Fournaise (AF), MSc Public Health, Industrial PhD-student, Region of Southern Denmark
Key In-country person: Søren Kabell Nissen (SKN), MD, PhD-student, Hospital of South West Jutland
Forward translation: Jessica Joan Williams (JJW), CNS, MSc in Public Health, University of Southern Denmark
Back translation: Claire Gudex (CG), MD, PhD, University of Southern Denmark
Proof-reading: Christina Boesen Kristensen (CBK), MA in Danish, Region of Southern Denmark
In-country consultant: Karen Andersen-Ranberg (KAR), Professor, MD, PhD, Odense University Hospital

Contact information: Anders Fournaise – anders.fournaise@rsyd.dk / Søren Kabell Nissen – sknissen@health.sdu.dk

Table of contents

Introduction 2
Study group 2
Table of contents 3
Detailed description of steps performed during the translation process 4
Step 1: Preparation 4
Step 2: Forward Translation 4
Step 3: Reconciliation 4
Step 4: Back-translation 4
Step 5: Back-translation review 4
Step 6: Harmonization 4
Step 7: Cognitive debriefing 5
Step 8: Review of cognitive debriefing results and finalization 5
Step 9: Proofreading 5
Step 10: Conclusions 5
Acknowledgements 6
Appendix 1 - Source instrument – Clinical Frailty Scale 2.0 7
Appendix 2 - Copyright permission 8
Appendix 3.1 - Forward-translation 1 9
Appendix 3.2 - Forward-translation 2 10
Appendix 4.1 - Reconciled version 12
Appendix 5 - Back-translation 13
Appendix 7 - Feedback from the designer of source instrument 15
Appendix 8.1 - Harmonized version 17
Appendix 9 - Final version 18

Contact information: Anders Fournaise – anders.fournaise@rsyd.dk / Søren Kabell Nissen – sknissen@health.sdu.dk

Detailed description of steps performed during the translation process

Step 1: Preparation

AF and SKN obtained permission to translate the CFS into the Danish language from the source instrument developer (appendix 1 and 2). The developer extended the previously obtained permission for the CFS 1.2 to the CFS 2.0.

In relation to the translation of the CFS version 1.2, the concept of frailty had already been investigated through a scoping review of relevant literature. Both the original development study (Ref: Rockwood 2005, CMAJ) and the study presenting the updated version of the Clinical Frailty Scale 2.0 (Ref: Rockwood 2020, CJG) was investigated in detail. AF and SKN then recruited researchers with key competences and informed them about the process to secure a coordinated and complete translation process (see members of the study group above). The study group was very familiar with the process after the recent translation of the CFS 1.2.

Step 2: Forward Translation

SKN (Danish native speaker, proficient in English, residing in DK) and JJW (English native speaker, proficient in Danish, residing in DK) independently completed a forward translation after AF had explained the background and concepts for the translation.

Step 3: Reconciliation

AF, SKN and KAR reconciled the two forward translations into a reconciled forward translation. Overall, the two forward translations had high agreement. The reconciliation for each item is described in detail in appendix 4.

Step 4: Back-translation

The back translation (appendix 5) was performed by CG, who is associate professor in health services research and a medical writer experienced in translation guidelines and instrument validation.

Step 5: Back-translation review

AF and SKN reviewed the back translation against the original Clinical Frailty Score (source instrument) to ensure conceptual equivalence of the translation. The back-translation was then sent to the developer of source instrument for revision and feedback. No major discrepancies were identified, and the reconciled translation was only slightly refined.
See appendix 6 for a detailed review of the back translation and appendix 7 for the feedback from the designer of source instrument.

Step 6: Harmonization

The minor changes proposed in the back-translation review were discussed in a harmonization meeting between AF, SKN and CG. An overview of the changes made to the reconciled translation is provided in appendix 8. The product of steps 3 and 5, the harmonized version, is presented in appendix 8.1.

Contact information: Anders Fournaise – anders.fournaise@rsyd.dk / Søren Kabell Nissen – sknissen@health.sdu.dk

Step 7: Cognitive debriefing

A cognitive debriefing was completed to explore alternative wording and to check for understandability, interpretation and cultural relevance of the translation.

For the cognitive debriefing, we asked eight health professionals (two senior consultants from a hospital geriatric department, two primary care physicians, two community nurses, and a consultant and senior consultant from a hospital intensive department) to complete three cases and provide feedback on the harmonized version of the CFS-DK 2.0. The assessment was completed using an online questionnaire built into REDCap (version: REDCap 9.1.15 - © 2020 Vanderbilt University). Redcap is an electronic data capture tool hosted at Open Patient data Exploratorive Network (OPEN) at Odense University Hospital, the Region of Southern Denmark.

Step 8: Review of cognitive debriefing results and finalization

The cognitive debriefing led to the following changes:

Level 3: But was replaced with a punctuation to better match usual length of sentences in the Danish language.

Level 4: "Tidligere "sårbare" ["previously vulnerable] was deleted as cognitive debriefer found the reference to the previous scale (CFS 1.2) confusing, looking for vulnerable in the CFS 2.0. This change was reported to the instrument developer.

Level 5: "Ofte" [Often] added and "som" [that] deleted to better reflect the prevalence of "slowing" in this group and increase correspondence with the source instrument.

Level 7: "()" around "inden for ca. 6 måneder" deleted.

Step 9: Proofreading

The final translation (appendix 9) was proofread by CBK who holds a Master of Sciences in Danish. This resulted in minor corrections of grammatical errors in level 2 and 6 and in the section describing the use of CFS in patients with dementia.

Step 10: Conclusions

To conclude the ISPOR translation process, SKN and AF drafted this final report, which was commented and approved by the study group. The translation process will be disseminated in an "correspondence" article, which will be submitted to BMC Geriatrics as a follow-up to the previously published translation and reliability test of CFS 1.2.

Contact information: Anders Fournaise – anders.fournaise@rsyd.dk / Søren Kabell Nissen – sknissen@health.sdu.dk

Acknowledgements

The study group wish to acknowledge Jens Vestergaard, Christel Kronborg, Katja Thomsen, Lars Matzen, one Lauritzen, Susan Feldborg, Nina Andersen-Ranberg and Ulf Pedersen for participating in the cognitive debriefing of the CFS-DK 2.0.

Finally, the authors also acknowledge David Hass from OPEN, Open Patient data Explorative Network, Odense University Hospital, Region of Southern Denmark for assistance in data management.

Contact information: Anders Fournaise – anders.fournaise@rsyd.dk / Søren Kabell Nissen – sknissen@health.sdu.dk

Appendix 1 - Source instrument – Clinical Frailty Scale 2.0

CLINICAL FRAILTY SCALE

1	VERY FIT	People who are robust, active, energetic and motivated. They tend to exercise regularly and are among the fittest for their age.
2	FIT	People who have **no active disease symptoms** but are less fit than category 1. Often, they exercise or are very **active occasionally**, e.g., seasonally.
3	MANAGING WELL	People whose **medical problems are well controlled**, even if occasionally symptomatic, but often are **not regularly active** beyond routine walking.
4	LIVING WITH VERY MILD FRAILTY	Previously "vulnerable," this category marks early transition from complete independence. While **not dependent** on others for daily help, often **symptoms limit activities**. A common complaint is being "slowed up" and/or being tired during the day.
5	LIVING WITH MILD FRAILTY	People who often have **more evident slowing**, and need help with **high order instrumental activities of daily living** (finances, transportation, heavy housework). Typically, mild frailty progressively impairs shopping and walking outside alone, meal preparation, medications and begins to restrict light housework.
6	LIVING WITH MODERATE FRAILTY	People who need help with **all outside activities** and with **keeping house**. Inside, they often have problems with stairs and need **help with bathing** and might need minimal assistance (cuing, standby) with dressing.
7	LIVING WITH SEVERE FRAILTY	**Completely dependent for personal care**, from whatever cause (physical or cognitive). Even so, they seem stable and not at high risk of dying (within ~6 months).
8	LIVING WITH VERY SEVERE FRAILTY	Completely dependent for personal care and approaching end of life. Typically, they could not recover even from a minor illness.
9	TERMINALLY ILL	Approaching the end of life. This category applies to people with a **life expectancy <6 months**, who are **not otherwise living with severe frailty**. (Many terminally ill people can still exercise until very close to death.)

SCORING FRAILTY IN PEOPLE WITH DEMENTIA

The degree of frailty generally corresponds to the degree of dementia. Common **symptoms in mild dementia** include forgetting the details of a recent event, though still remembering the event itself, repeating the same question/story and social withdrawal.

In **moderate dementia**, recent memory is very impaired, even though they seemingly can remember their past life events well. They can do personal care with prompting.

In **severe dementia**, they cannot do personal care without help.

In **very severe dementia** they are often bedfast. Many are virtually mute.

DALHOUSIE UNIVERSITY

Clinical Frailty Scale ©2005–2020 Rockwood, Version 2.0 (EN). All rights reserved. For permission: www.geriatricmedicineresearch.ca

Rockwood K et al. A global clinical measure of fitness and frailty in elderly people. CMAJ 2005;173:489–495.

Contact information: Anders Fournaise – anders.fournaise@rsyd.dk / Søren Kabell Nissen – sknissen@health.sdu.dk

Appendix 2 - Copyright permission

DALHOUSIE UNIVERSITY

FOR OFFICE USE ONLY
PF#: 20200316-05

PERMISSION TO USE THE CLINICAL FRAILTY SCALE (CFS)©

The undersigned is granted permission to use, reproduce and distribute the Clinical Frailty Scale (CFS)© in the format attached[1] for educational purposes and for non-commercially funded research and/or quality assurance projects. The CFS© must be administered free of charge to patients and/or study participants.

A formal Licensing Agreement is required for research funded by any commercial entity or pharma and, in some cases, for use in routine clinical care.

The copyright holder reserves the right to prospectively follow-up at any time to determine whether use of the CFS© meets the conditions described above.

Reselling of the CFS© or other commercial development without a license agreement is prohibited by copyright.

The undersigned, their delegates and affiliated organization(s) agree that they will not claim ownership rights to the CFS©, or any derivative, including translations, compilation, sequel or series. Nothing in this Agreement shall give the undersigned any right, title, or interest in the CFS© other than the right to use in accordance with this Agreement. The CFS© will not be modified unless explicitly given permission to do so.

**USER INFORMATION:**	
Full Name:	Soren Kabell Nissen
Position/Title:	MD, PhD student
Institution/Organization:	Institute for Health Research, University of Southern Denmark
Mailing Address:	Finsensgade 35, 6700 Esbjerg, Denmark
Telephone:	+45 21 80 38 17
Email:	SKNissen@health.sdu.dk
Type of organization:	☐ For-profit ☒ Not-for-profit ☐ Other, *please specify:*

INTENDED USE (Select all that apply):

☐ Reprint — *Provide publication details:*

☒ Research study / clinical trial — *Describe use in study:* 1. Perform translation to Danish according to the standards suggested by the ISPOR Task Force for Translation and Cultural Adaptation. 2. Validate in Danish primary care setting as part of a randomized controlled clinical trial.
Expected duration of study: Start date 04.2020 End date 05.2021

☐ Routine clinical care
Will the CFS© be incorporated into an electronic medical health record (EMR)? ☐ Y ☐ N
***If yes**, please identify whether an EMR company is involved (e.g., Epic, Meditech):*

☐ Other — *Specify:*

Are you planning to translate the CFS©? ☒ Y ☐ N ***If yes**, specify language(s):* Danish
We request editable (e.g. MSWord) copies of all translations. We do not independently verify or validate translations.

Are you planning any commercial development that would incorporate the CFS©?
☐ Y ☒ N ***If yes**, please specify:*

By your signature below, you attest that you understand the conditions under which permission is granted.

Signature: [signature]
Date: 03.11.2020

Send completed and signed form by post/fax or email to:
Kenneth Rockwood, MD
1421-5955 Veterans' Memorial Lane
Halifax, NS B3H 2E1 Canada
Fax: 1-902-473-1050 | Email: gmru@dal.ca

Approved by: [signature] Date: 2020/03/16
Kenneth Rockwood, MD (or delegate)

[1] A copy of the CFS will be emailed to the user upon review and approval of this permission form. Valid only when signed by all parties.

CFS© Permission For Use Form V6 2019Sep18

Contact information: Anders Fournaise – anders.fournaise@rsyd.dk / Søren Kabell Nissen – sknissen@health.sdu.dk

Appendix 3.1 - Forward-translation 1

Performed by JJW

1: Meget god form – Mennesker der er robuste, aktive, energiske og motiverede. Typisk motionerer de regelmæssigt og er blandt dem i bedst form for deres alder.
2: God form – Mennesker uden aktive symptomer på sygdom, men i mindre god form end kategori 1. De motionerer ofte eller er meget aktive en gang imellem, f.eks. på bestemte årstider.
3: Klarer sig godt – Mennesker<br>med velkontrollerede sygdomsproblemer, selvom de lejlighedsvis er symptomatiske, men som ofte ikke er regelmæssigt aktive udover rutinemæssige gåture.
4: Meget mildt skrøbelighed – Tidligere kaldt "Sårbar", denne kategori markerer den begyndende overgang fra total uafhængighed. Mennesker der ikke er afhængige af andre til daglige gøremål, men som ofte har symptomer, der begrænser aktiviteterne. En almindelig klage er at føle sig "langsom" eller træt i løbet af dagen.
5: Mildt skrøbelighed – Mennesker der er mere tydeligt langsomme, og som har behov for hjælp til komplekse daglige gøremål (IADL - økonomi, transport, hovedrengøring). Typisk vil mild skrøbelighed i stigende grad hæmme indkøb, gåture alene udenfor, madlavning, medikamenter og let husarbejde.
6. Moderat skrøbelighed – Mennesker der har behov for hjælp til alle udendørs aktiviteter og med at holde hus. Indendørs har de ofte problemer med trappegang, hjælp til at gå i bad og kan nogle gange have brug for minimal hjælp til påklædning (stikord, let støtte ved behov).
7: Svært skrøbelighed – Fuldstændig afhængige af hjælp til egenomsorg, uanset årsag (fysisk eller kognitiv). Alligevel virker de stabile og ikke i høj risiko for at dø (indenfor ca. 6 måneder).
8: Meget svært skrøbelighed – Fuldstændig afhængige af hjælp til egenomsorg og nærmer sig livets afslutning. Typisk vil de ikke engang komme sig efter let sygdom.
9: Terminalt syg – Mennesker der nærmer sig livets afslutning, men ikke fremstår tydeligt skrøbelige i øvrigt. (Mange terminalt syge mennesker formår stadig at motionere ind til tæt på dødens indtræden). Denne kategori gælder mennesker med en forventet levetid på mindre 6 måneder.
Bedømmelse af skrøbelighed hos mennesker med demens
Mennesker med demens er skrøbelige (scorer altid minimum 5) og graden af skrøbelighed svarer generalt til graden af demens. Typiske symptomer ved mild demens er at glemme detaljer om en nylig begivenhed, selvom man kan huske selve begivenheden, og at gentage det samme spørgsmål/historie og social tilbagetrækning.
Ved moderat demens er hukommelsen for nylige begivenheder svært nedsat, selvom man tilsyneladende kan huske gamle minder tydeligt. Man kan udføre personlig pleje med vejledning.
Ved svær demens kan man ikke udføre personlig pleje uden hjælp.
Ved meget svær demens er man ofte sengeliggende. Mange er stort set stumme.
Clinical Frailty Scale © 2005-2020 Rockwood, Version 2.0 (EN). All rights reserved. For permission: www.geriatricmedicineresearch.ca Rockwood K et al. A global clinical measure of fitness and frailty in elderly people. CMAJ 2005:173;489-495.

Contact information: Anders Fournaise – anders.fournaise@rsyd.dk / Søren Kabell Nissen – sknissen@health.sdu.dk

Appendix 3.2 - Forward-translation 2

Performed by SKN

1: **Meget god form** – Mennesker der er robuste, aktive, energiske og motiverede. De motionerer typisk regelmæssigt og er blandt dem i bedst form for deres alder.
2: **God form** – Mennesker **uden aktive symptomer på sygdom**, men i mindre god form end kategori 1. De motionerer ofte eller er meget **aktive en gang imellem**, f.eks. på bestemte årstider.
3: **Klarer sig godt** – Mennesker med **velkontrollerede sygdomsproblemer** selvom de af og til har symptomer, men oftest **ikke regelmæssigt aktive** udover rutinemæssige gåture.
4: **Lever med meget mild skrøbelighed** – Tidligere ”sårbare”. Denne kategori markerer starten på overgangen fra komplet uafhængighed. Mennesker der **ikke er afhængige** af andre til daglige gøremål, men som ofte har **symptomer, der begrænser aktiviteterne**. En almindelig klage er at føle sig ”langsom” eller træt i løbet af dagen.
5: **Lever med mild skrøbelighed** – Mennesker der er **mere tydeligt langsomme**, og som har **behov for hjælp til komplekse daglige gøremål (***Instrumental Activities of Daily Living* – økonomi, transport, hovedrengøring). Typisk vil mild skrøbelighed i stigende grad hæmme indkøb, gåture alene udenfor, madlavning, medicin og begynde at begrænse let husarbejde.
6: **Lever med moderat skrøbelighed** – Mennesker med behov for hjælp til **alle udendørs aktiviteter** og med at **holde hus**. Indendørs har de ofte problemer med trappegang, **hjælp til at gå i bad** og kan nogle gange have brug for minimal hjælp til påklædning (stikord, let støtte ved behov).
7: **Lever med svær skrøbelighed** – **Fuldstændig afhængige af hjælp til egenomsorg**, uanset årsag (fysisk eller kognitiv). Alligevel virker de stabile og ikke i høj risiko for at dø (indenfor ca. 6 måneder).
8: **Lever med meget svær skrøbelighed** – Fuldstændig afhængige af hjælp til personlig pleje og nærmer sig livets afslutning. Typisk vil de ikke engang komme sig efter let sygdom.
9: **Terminalt syg** – Mennesker som nærmer sig livets afslutning, men **ikke i øvrigt lever med meget svær skrøbelighed**. Denne kategori gælder mennesker med en **forventet levetid på mindre 6 måneder**. (Mange terminalt syge mennesker kan stadig motionere helt indtil livets afslutning)
**Bedømmelse af skrøbelighed hos mennesker med demens**
Mennesker med demens er skrøbelige (scorer altid minimum 5) og graden af skrøbelighed svarer ofte til graden af demens. Typiske **symptomer ved mild demens** er at glemme detaljer om en nylig begivenhed, selvom man kan huske selve begivenheden, og at gentage det samme spørgsmål/historie og social tilbagetrækning.
Ved **moderat demens** er hukommelsen for nylige begivenheder svært nedsat, selvom man tilsyneladende kan huske gamle minder tydeligt. Man kan udføre personlig pleje med vejledning.
Ved **svær demens** kan man ikke udføre personlig pleje uden hjælp.
Ved **meget svær demens** er man ofte sengeliggende. Mange har nærmest mistet evnen til at tale.
Clinical Frailty Scale © 2005-2020 Rockwood, Version 2.0 (EN). All rights reserved. For permission: www.geriatricmedicineresearch.ca<br>Rockwood K et al. A global clinical measure of fitness and frailty in elderly people. CMAJ 2005:173;489-495.<br>Danish Version 2.0, 2020, translated by Anders Fournaise and Søren Kabell Nissen, University of Southern Denmark.

Contact information: Anders Fournaise – anders.fournaise@rsyd.dk / Søren Kabell Nissen – sknissen@health.sdu.dk

Appendix 4 - Reconciliation

Performed by SKN, KAR and AF

Item 1 decisions:

- “De” instead of “Mennesker” was chosen.

Item 3 decisions:

- “indimellem” was chosen instead of “lejlighedsvise” and “af og til”.

Item 4 decisions:

- “en begyndende overgang” was chosen instead of “starten på overgangen”.
- “komplet” was chosen over “total”

Item 5 decisions:

- “begynde at begrænse let husarbejde” was chosen over ”let husarbejde”.

Item 8 decisions:

- “personlig pleje” was chosen instead of “egen omsorg”.

Item 9 decisions:

- The sentence construct in SKN translation was chosen as it better fits the Danish language.
- “kan” was chosen instead of “formår”.

Item on dementia, decisions:

- “ofte” was chosen instead of “generalt”
- “Mange har nærmest mistet evnen til at tale” was chosen over ”mange er stort set stumme”.

Contact information: Anders Fournaise – anders.fournaise@rsyd.dk / Søren Kabell Nissen – sknissen@health.sdu.dk

Appendix 4.1 - Reconciled version

1: **Meget god form** – Mennesker der er robuste, aktive, energiske og motiverede. De motionerer typisk regelmæssigt og er blandt dem i bedst form for deres alder.
2: **God form** – Mennesker **uden aktive symptomer på sygdom**, men i mindre god form end kategori 1. De motionerer ofte eller er meget **aktive en gang imellem**, f.eks. på bestemte årstider.
3: **Klarer sig godt** – Mennesker med **velkontrollerede sygdomsproblemer**, selvom de indimellem har symptomer, men oftest **ikke regelmæssigt aktive** udover rutinemæssige gåture.
4: **Lever med meget mild skrøbelighed** – Tidligere "sårbare". Denne kategori markerer en begyndende overgang fra komplet uafhængighed. Mennesker der **ikke er afhængige** af andre til daglige gøremål, men som ofte har **symptomer, der begrænser aktiviteterne**. En almindelig klage er at føle sig "langsom" eller træt i løbet af dagen.
5: **Lever med mild skrøbelighed** – Mennesker der er **mere tydeligt langsomme**, og som har **behov for hjælp til komplekse daglige gøremål** (*Instrumental Activities of Daily Living* – økonomi, transport, hovedrengøring). Typisk vil mild skrøbelighed i stigende grad hæmme indkøb, gåture alene udenfor, madlavning, medicin og begynde at begrænse let husarbejde.
6: **Lever med moderat skrøbelighed** – Mennesker der har behov for hjælp til **alle udendørs aktiviteter** og med at **holde hus**. Indendørs har de ofte problemer med trappegang, **hjælp til at gå i bad** og kan nogle gange have brug for minimal hjælp til påklædning (stikord, let støtte ved behov).
7: **Lever med svær skrøbelighed** – **Fuldstændig afhængige af hjælp til egenomsorg**, uanset årsag (fysisk eller kognitiv). Alligevel virker de stabile og ikke i høj risiko for at dø (indenfor ca. 6 måneder).
8: **Lever med meget svær skrøbelighed** – Fuldstændig afhængige af hjælp til personlig pleje og nærmer sig livets afslutning. Typisk vil de ikke engang komme sig efter let sygdom.
9: **Terminalt syg** – Mennesker der nærmer sig livets afslutning. Denne kategori gælder mennesker med en **forventet levetid på mindre 6 måneder**, som **ikke lever med meget svær skrøbelighed i øvrigt**. (Mange terminalt syge mennesker kan stadig motionere helt indtil livets afslutning)
**Bedømmelse af skrøbelighed hos mennesker med demens**
Mennesker med demens er skrøbelige (scorer altid minimum 5) og graden af skrøbelighed svarer ofte til graden af demens. Typiske **symptomer ved mild demens** er at glemme detaljer om en nylig begivenhed, selvom man kan huske selve begivenheden, og at gentage det samme spørgsmål/historie og social tilbagetrækning.
Ved **moderat demens** er hukommelsen for nylige begivenheder svært nedsat, selvom man tilsyneladende kan huske gamle minder tydeligt. Man kan udføre personlig pleje med vejledning.
Ved **svær demens** kan man ikke udføre personlig pleje uden hjælp.
Ved **meget svær demens** er man ofte sengeliggende. Mange har nærmest mistet evnen til at tale.
Clinical Frailty Scale © 2005-2020 Rockwood, Version 2.0 (EN). All rights reserved. For permission: www.geriatricmedicineresearch.ca<br>Rockwood K et al. A global clinical measure of fitness and frailty in elderly people. CMAJ 2005:173;489-495.<br>Danish Version 2.0, 2020, translated by Anders Fournaise and Søren Kabell Nissen, University of Southern Denmark.

Contact information: Anders Fournaise – anders.fournaise@rsyd.dk / Søren Kabell Nissen – sknissen@health.sdu.dk

Appendix 5 - Back-translation

Performed by CG

1: **Very fit** - People who are robust, active, energetic and motivated. They typically exercise regularly and are among the fittest for their age.
2: **Fit** - People **without active symptoms of illness** but not as fit as category 1. They exercise often or are very **active once in a while**, e.g. at certain times of the year.
3: **Manage well** - People with **well-controlled medical conditions**, even if they occasionally have symptoms, but are often not regularly active apart from routine walks.
4: **Living with very mild frailty** – Previously 'vulnerable'. This category marks the start of a transition from complete independence. People who are **not dependent** on others for daily activities but often have symptoms that restrict their activities. A common complaint is feeling "slow" or tired during the day.
5: **Living with mild frailty** - People who are **more clearly slow** and **need help with complex daily activities** (*Instrumental Activities of Daily Living* - finances, transport, heavy housework). Typically, mild frailty will increasingly limit shopping, walking alone outside, cooking, medicine/medications and will begin to restrict light housework.
6: **Living with moderate frailty** - People who need help with **all outdoor activities** and with **housekeeping**. Indoors, they often have trouble with stairs, **need help with bathing** and sometimes minimal help with dressing (prompts, helping hand when needed).
7: **Living with severe frailty** - **Fully dependent on others for personal care**, for whatever reason (physical or cognitive). However, they appear stable and not at high risk of dying (within about 6 months).
8: **Living with very severe frailty** – Completely dependent on help with personal care and nearing the end of life. They will typically not recover from even slight illness.
9: **Terminally ill** – People who are nearing the end of life. This category refers to people with a **life expectancy of less than 6 months** but who otherwise **do not live with very severe frailty**. (Many terminally ill people can still exercise until the very end of life).
**Assessing frailty in people with dementia**
People with dementia are frail (always score at least 5), and the degree of frailty often corresponds to the degree of dementia. Typical **symptoms of mild dementia** are forgetting the details about a recent event although remembering the event itself, and repeating the same question/story and social withdrawal.
People with **moderate dementia** have severely impaired memory of recent events even though they seem to remember old memories clearly. They can perform personal care with guidance.
People with **severe dementia** cannot perform personal care without help.
People with **very severe dementia** are often confined to bed. Many have nearly lost the ability to speak.
Clinical Frailty Scale © 2005-2020 Rockwood, Version 2.0 (EN). All rights reserved. For permission: www.geriatricmedicineresearch.ca<br>Rockwood K et al. A global clinical measure of fitness and frailty in elderly people. CMAJ 2005:173;489-495.

Contact information: Anders Fournaise – anders.fournaise@rsyd.dk / Søren Kabell Nissen – sknissen@health.sdu.dk

Appendix 6 - Back-translation review

Performed by AF and SKN

No omissions or obvious mistranslations were detected. A point-by-point list is presented below.

Item 1

- No significant conceptual discrepancies

Item 2

- A minor discrepancy in the use of the word 'often'. This issue was not resolved in the back-translation review as it required discussion with the back-translator (CG)

Item 3

- No significant conceptual discrepancies, not necessary to refine translation.

Item 4

- No significant conceptual discrepancies, not necessary to refine translation.

Item 5

- No significant conceptual discrepancies, not necessary to refine translation.

Item 6

- Similarly to item 2 the place of the word often need to be discussed in the harmonization meeting.

Item 7

- No significant conceptual discrepancies, not necessary to refine translation

Item 8

- No significant conceptual discrepancies, not necessary to refine translation

Item 9

- No significant conceptual discrepancies, not necessary to refine translation

Item on dementia

- No significant conceptual discrepancies, not necessary to refine translation

Contact information: Anders Fournaise – anders.fournaise@rsyd.dk / Søren Kabell Nissen – sknissen@health.sdu.dk

Appendix 7 - Feedback from the designer of source instrument

RE: CFS 2.0 in Danish translation

Olga Theou <Olga.Theou@Dal.Ca>
to 17-12, 22:38
Anders Fournaise; Søren Kabell Nissen <sknissen@health.sdu.dk>; +2 flere

Besvar

Indbakke

Du svarede den 18-12-2020 11:49.

Dear Anders,
I am sorry for the delayed reply. We now reviewed all the documents you provided. You did a great job with all the steps of this project and with the documentation of the process. We only have one comment. When we made the last changes in the CFS we wanted to emphasize that it Is true that most people with dementia are frail but there are some people with early stages of dementia who are fit and may be scored less than 5. For this reason I would recommend the following changes to your text "People with dementia are often frail (most score at least 5), and the degree of frailty often corresponds to the degree of dementia."

Let me know if you have any additional questions

Olga

Olga Theou, PhD
Canada Research Chair in Physical Activity, Mobility, and Healthy Aging
Assistant Professor, Physiotherapy and Geriatric Medicine, Dalhousie University
Affiliated Scientist, Geriatric Medicine, Nova Scotia Health Authority
Adjunct Senior Lecturer, School of Medicine, University of Adelaide

Contact information: Anders Fournaise – anders.fournaise@rsyd.dk / Søren Kabell Nissen – sknissen@health.sdu.dk

Appendix 8 - Harmonization
Participants: AF, SKN and CG

Item 1

- Conceptual discrepancy was detected regarding the meaning of ‘often’. Do these people exercise often? - or are they often people who exercise or are very active once in a while? The later was chosen as it corresponds with the source instrument.

Item 2

- No changes added

Item 3

- No changes added

Item 4

- No changes added

Item 5

- No changes added

Item 6

- Similarly to item 2 the place of the word often was moved to the beginning of the sentence.

Item 7

- No changes added

Item 8

- No changes added

Item 9

- No changes added

Item on dementia

- As for the CFS 1.2, we added “Mennesker med demens er oftest skrøbelige (scorer oftest minimum 5)”. The logical implication that CFS will often be 5 or above for people with dementia needed to be concretized, this might reduce misinterpretation and increase correspondence with the source tool. This change was discussed with and accepted by the source instrument developer.

Contact information: Anders Fournaise – anders.fournaise@rsyd.dk / Søren Kabell Nissen – sknissen@health.sdu.dk

SDU University of Southern Denmark Region of Southern Denmark

Appendix 8.1 - Harmonized version

1: **Meget god form** – Mennesker der er robuste, aktive, energiske og motiverede. De motionerer typisk regelmæssigt og er blandt dem i bedst form for deres alder.
2: **God form** – Mennesker uden aktive symptomer på sygdom, men i mindre god form end kategori 1. Ofte motionerer de eller er meget aktive en gang imellem, f.eks. på bestemte årstider.
3: **Klarer sig godt** – Mennesker med velkontrollerede sygdomsproblemer, selvom de indimellem har symptomer, men oftest ikke regelmæssigt aktive udover rutinemæssige gåture.
4: **Lever med meget mild skrøbelighed** – Tidligere ”sårbare”. Denne kategori markerer en begyndende overgang fra komplet uafhængighed. Mennesker der ikke er afhængige af andre til daglige gøremål, men som ofte har symptomer, der begrænser aktiviteterne. En almindelig klage er at føle sig ”langsom” eller træt i løbet af dagen.
5: **Lever med mild skrøbelighed** – Mennesker der er mere tydeligt langsomme, og som har behov for hjælp til komplekse daglige gøremål (*Instrumental Activities of Daily Living* – økonomi, transport, hovedrengøring). Typisk vil mild skrøbelighed i stigende grad hæmme indkøb, gåture alene udenfor, madlavning, medicin og begynde at begrænse let husarbejde.
6: **Lever med moderat skrøbelighed** – Mennesker der har behov for hjælp til alle udendørs aktiviteter og med at holde hus. Ofte har de problemer med indendørs trappegang og behøver hjælp til at gå i bad, og kan eventuelt have brug for minimal hjælp til påklædning (stikord, let støtte ved behov).
7: **Lever med svær skrøbelighed** – Fuldstændig afhængige af hjælp til egenomsorg, uanset årsag (fysisk eller kognitiv). Alligevel virker de stabile og ikke i høj risiko for at dø (indenfor ca. 6 måneder).
8: **Lever med meget svær skrøbelighed** – Fuldstændig afhængige af hjælp til personlig pleje og nærmer sig livets afslutning. Typisk vil de ikke engang komme sig efter let sygdom.
9: **Terminalt syg** – Mennesker der nærmer sig livets afslutning. Denne kategori gælder mennesker med en forventet levetid på mindre end 6 måneder, som ikke lever med svær skrøbelighed i øvrigt. (Mange terminalt syge mennesker kan stadig motionere helt indtil livets afslutning)
Bedømmelse af skrøbelighed hos mennesker med demens
Mennesker med demens er oftest skrøbelige (scorer oftest minimum 5) og graden af skrøbelighed svarer som regel til graden af demens. Typiske symptomer ved mild demens er at glemme detaljer om en nylig begivenhed, selvom man kan huske selve begivenheden, og at gentage det samme spørgsmål/historie og social tilbagetrækning.
Ved **moderat demens** er hukommelsen for nylige begivenheder svært nedsat, selvom man kan huske gamle minder tydeligt. Man kan udføre personlig pleje med vejledning.
Ved **svær demens** kan man ikke udføre personlig pleje uden hjælp.
Ved **meget svær demens** er man ofte sengeliggende. Mange er nærmest ophørt med at tale.
Clinical Frailty Scale © 2005-2020 Rockwood, Version 2.0 (EN). All rights reserved. For permission: www.geriatricmedicineresearch.ca<br>Rockwood K et al. A global clinical measure of fitness and frailty in elderly people. CMAJ 2005:173;489-495.<br>Danish Version 2.0, 2020, translated by Anders Fournaise and Søren Kabell Nissen, University of Southern Denmark.
1: **Meget god form** – Mennesker der er robuste, aktive, energiske og motiverede. De motionerer typisk regelmæssigt og er blandt dem i bedst form for deres alder.

Contact information: Anders Fournaise – anders.fournaise@rsyd.dk / Søren Kabell Nissen – sknissen@health.sdu.dk

2: **God form** – Mennesker uden aktive symptomer på sygdom, men i mindre god form end kategori 1. Ofte motionerer de eller er meget aktive en gang imellem, f.eks. på bestemte årstider.

Appendix 9 - Final version

CLINICAL FRAILTY SCALE

1 MEGET GOD FORM – Mennesker der er robuste, aktive, energiske og motiverede. De motionerer typisk regelmæssigt og er blandt dem i bedst form for deres alder.

2 GOD FORM – Mennesker **uden aktive symptomer på sygdom**, men i mindre god form end kategori 1. Ofte motionerer de eller er meget **aktive en gang imellem**, f.eks. på bestemte årstider.

3 KLARER SIG GODT – Mennesker med **velkontrollerede sygdomsproblemer**, selvom de indimellem har symptomer. Oftest er de **ikke regelmæssigt aktive** udover rutinemæssige gåture.

4 LEVER MED MEGET MILD SKRØBELIGHED – Denne kategori markerer en begyndende overgang fra komplet uafhængighed. Mennesker der **ikke er afhængige** af andre til daglige gøremål, men som ofte har **symptomer, der begrænser aktiviteterne**. En almindelig klage er at føle sig "langsom" eller træt i løbet af dagen.

5 LEVER MED MILD SKRØBELIGHED – Mennesker der ofte er **mere tydeligt langsomme**, og har behov for hjælp til **komplekse daglige gøremål** (*Instrumental Activities of Daily Living* – økonomi, transport, hovedrengøring). Typisk vil mild skrøbelighed i stigende grad hæmme indkøb, gåture alene udenfor, madlavning, medicin og begynde at begrænse let husarbejde.

6 LEVER MED MODERAT SKRØBELIGHED – Mennesker der har behov for hjælp til **alle udendørs aktiviteter** og med **at holde hus**. Ofte har de problemer med indendørs trappegang og behøver **hjælp til at gå i bad** og kan eventuelt have brug for minimal hjælp til påklædning (stikord, let støtte ved behov).

7 LEVER MED SVÆR SKRØBELIGHED – **Fuldstændig afhængige af hjælp til egenomsorg**, uanset årsag (fysisk eller kognitiv). Alligevel virker de stabile og ikke i høj risiko for at dø inden for ca. 6 måneder.

8 LEVER MED MEGET SVÆR SKRØBELIGHED – Fuldstændig afhængige af hjælp til personlig pleje og nærmer sig livets afslutning. Typisk vil de ikke engang komme sig efter let sygdom.

9 TERMINALT SYG – Mennesker der nærmer sig livets afslutning. Denne kategori gælder mennesker med en **forventet levetid på mindre 6 måneder**, som **ikke lever med svær skrøbelighed i øvrigt** (Mange terminalt syge mennesker kan stadig motionere helt indtil livets afslutning).

BEDØMMELSE AF SKRØBELIGHED HOS MENNESKER MED DEMENS

Mennesker med demens er oftest skrøbelige (scorer oftest minimum 5) og graden af skrøbelighed svarer som regel til graden af demens. Typiske **symptomer ved mild demens** er at glemme detaljer om en nylig begivenhed, selvom man kan huske selve begivenheden og at gentage det samme spørgsmål/historie og social tilbagetrækning.

Ved **moderat demens** er hukommelsen for nylige begivenheder svært nedsat, selvom man kan huske gamle minder tydeligt. Man kan udføre personlig pleje med vejledning.

Ved **svær demens** kan man ikke udføre personlig pleje uden hjælp.

Ved **meget svær demens** er man ofte sengeliggende. Mange er nærmest ophørt med at tale.

DALHOUSIE UNIVERSITY

Clinical Frailty Scale © 2005-2020 Rockwood, Version 2.0 (EN). All rights reserved. For permission: www.geriatricmedicineresearch.ca
Rockwood K et al. A global clinical measure of fitness and frailty in elderly people. CMAJ 2005;173:489-495.
Danish Version 2.0, 2020, translated by Anders Fournaise and Søren Kabell Nissen, University of Southern Denmark.

Contact information: Anders Fournaise – anders.fournaise@rsyd.dk / Søren Kabell Nissen – sknissen@health.sdu.dk
