# Supplementary material for: Translation of the updated clinical frailty scale 2.0 into Danish and implications for cross-sectoral reliability
Source: BMC Geriatr. 2021 Apr 21;21:269. doi: 10.1186/s12877-021-02222-w (PMC8059120; doi:10.1186/s12877-021-02222-w)
Supplement: Supplementary file 2 — Additional file 2. Clinical Frailty Scale version 1.2 and 2.0 in English and Danish, with the differences between the 1.2 and 2.0 versions highlighted. [file 12877_2021_2222_MOESM2_ESM.pdf]

Translation of the updated Clinical Frailty Scale 2.0 into Danish and implications for cross-sectoral reliability

Anders Fournaise (ORCID - 0000-0002-4754-7500)* [1,2,3], Søren Kabell Nissen (ORCID - 0000-0003-3722-908X) * [4,5], Jørgen T. Lauridsen (ORCID - 0000-0001-9889-6236) [6], Jesper Ryg (ORCID - 0000-0002-8641-3062) [2,9], Christian H. Nickel (ORCID - 0000-0001-6619-9284) [4,8], Claire Gudex (ORCID - 0000-0003-3881-9890) [9,10], Mikkel Brabrand (ORCID - 0000-0002-3340-8251) [4,5,7,9], Lone Musaeus Poulsen (ORCID - 0000-0002-7030-3395) [11,12], Karen Andersen-Ranberg (ORCID - 0000-0003-1970-7076) [2,3,9]

[1] Department of Cross-sectoral Collaboration, Region of Southern Denmark, 7100 Vejle, Denmark

[2] Department of Geriatric Medicine, Odense University Hospital, 5000 Odense, Denmark

[3] Epidemiology, Biostatistics and Biodemography, Department of Public Health, University of Southern Denmark, 5000 Odense, Denmark

[4] Institute of Regional Health Research, Centre South West Jutland, University of Southern Denmark, 6700 Esbjerg, Denmark

[5] Department of Emergency Medicine, Hospital of South West Jutland, 6700 Esbjerg, Denmark

[6] Department of Business and Economics, University of Southern Denmark, 5230 Odense, Denmark

[7] Department of Emergency Medicine, Odense University Hospital, 5000 Odense, Denmark

[8] Emergency Department, University Hospital Basel, University of Basel, 4031 Basel, Switzerland

[9] Department of Clinical Research, University of Southern Denmark, 5000 Odense, Denmark

[10] Open Patient data Explorative Network (OPEN), Region of Southern Denmark, 5000 Odense, Denmark

[11] Department of Anaesthesiology, Zealand University Hospital, Koege, Denmark

[12] Collaboration for Research in Intensive Care (CRIC), Copenhagen, Denmark

*co-first authors

Corresponding author

Anders Fournaise, MSPH

Department of Cross-sectoral Collaboration, Region of Southern Denmark, Damhaven 12, 7100 Vejle, Denmark

E-mail: anders.fournaise@rsyd.dk / 0045 2482 4315

Additional file 2. Clinical Frailty Scale version 1.2 and 2.0 in English and Danish, with the differences between the 1.2 and 2.0 versions highlighted

**CFS 1.2**	**CFS 2.0**	**CFS-DK 1.2**	**CFS-DK 2.0**
**Clinical Frailty Scale***	**Clinical Frailty Scale**	**Clinical Frailty Scale***	**Clinical Frailty Scale**
1 Very Fit – People who are robust, active, energetic and motivated. These people commonly exercise regularly. They are among the fittest for their age.	1 Very Fit – People who are robust, active, energetic and motivated. **They tend to** exercise regularly **and** are among the fittest for their age.	1: Meget god form – Mennesker der er robuste, aktive, energiske og motiverede. Typisk motionerer disse mennesker regelmæssigt. De er blandt dem i bedst form for deres alder.	1: Meget god form – Mennesker der er robuste, aktive, energiske og motiverede. **De** motionerer typisk regelmæssigt og er blandt dem i bedst form for deres alder.
2 Well – People who have no active disease symptoms but are less fit than category 1. Often they exercise or are very active occasionally, e.g. seasonally.	2 **Fit** – People who have no active disease symptoms but are less fit than category 1. Often they exercise or are very active occasionally, e.g. seasonally.	2: Velbefindende – Mennesker uden aktive symptomer på sygdom, men i mindre god form end kategori 1. De motionerer ofte eller er meget aktive en gang imellem, f.eks. på bestemte årstider.	2: **God form** – Mennesker uden aktive symptomer på sygdom, men i mindre god form end kategori 1. **Ofte** motionerer de eller er meget aktive en gang imellem, f.eks. på bestemte årstider.
3 Managing Well – People whose medical problems are well controlled, but are not regularly active beyond routine walking.	3 Managing Well – People whose medical problems are well controlled, **even if occasionally symptomatic,** but **often** are not regularly active beyond routine walking.	3: Klarer sig godt – Mennesker med velkontrollerede sygdomsproblemer, men som ikke er regelmæssigt aktive udover rutinemæssige gåture.	3: Klarer sig godt – Mennesker med velkontrollerede sygdomsproblemer, **selvom de indimellem har symptomer. Oftest er de** ikke regelmæssigt aktive udover rutinemæssige gåture.
4 Vulnerable – While not dependent on others for daily help, often symptoms limit activities. A common complaint is being "slowed up", and/or being tired during the day.	4 **Living with Very Mild Frailty – Previously "vulnerable", this category marks early transition from complete independence.** While not dependent on others for daily help, often symptoms limit activities. A common complaint is being "slowed up" and/or being tired during the day.	4: Sårbar – Mennesker der ikke er afhængige af andre til daglige gøremål, men som ofte har symptomer, der begrænser aktiviteterne. En almindelig klage er at føle sig "langsom" eller træt i løbet af dagen.	4: **Lever med meget mild skrøbelighed – Denne kategori markerer en begyndende overgang fra komplet uafhængighed.** Mennesker der ikke er afhængige af andre til daglige gøremål, men som ofte har symptomer, der begrænser aktiviteterne. En almindelig klage er at føle sig "langsom" eller træt i løbet af dagen.
5 Mildly Frail – These people often have more evident slowing, and need help in high order IADLs (finances, transportation, heavy housework, medications). Typically, mild frailty progressively impairs shopping and walking outside alone, meal preparation and housework.	5 **Living with Mild Frailty** – **People who** often have more evident slowing, and need help **with** high order **instrumental activities of daily living** (finances, transportation, heavy housework). Typically, mild frailty progressively impairs shopping and walking outside alone, meal preparation, **medications** and **begins to restrict light** housework.	5: Mildt skrøbelig – Mennesker der er mere tydeligt langsomme, og som har behov for hjælp til komplekse daglige gøremål (IADL - økonomi, transport, hovedrengøring, medicin). Typisk vil mild skrøbelighed i stigende grad hæmme indkøb, gåture alene udenfor, madlavning og husarbejde.	5: **Lever med mild skrøbelighed** – Mennesker der **ofte** er mere tydeligt langsomme, og har behov for hjælp til komplekse daglige gøremål **(Instrumental Activities of Daily Living** – økonomi, transport, hovedrengøring). Typisk vil mild skrøbelighed i stigende grad hæmme indkøb, gåture alene udenfor, madlavning, **medicin** og **begynde at begrænse let** husarbejde.
6 Moderately Frail – People need help with all outside activities and with keeping house. Inside, they often have problems with stairs and need help with bathing and might need minimal assistance (cuing, standby) with dressing.	6 **Living with Moderate Frailty** – People **who** need help with all outside activities and with keeping house. Inside, they often have problems with stairs and need help with bathing and might need minimal assistance (cuing, standby) with dressing.	6: Moderat skrøbelig – Mennesker med behov for hjælp til alle udendørs aktiviteter og med at holde hus. Indendørs har de ofte problemer med trappegang, hjælp til at gå i bad og kan nogle gange have brug for minimal hjælp til påklædning (stikord, let støtte ved behov).	6: **Lever med moderat skrøbelighed** – Mennesker **der har** behov for hjælp til alle udendørs aktiviteter og med at holde hus. **Ofte** har de problemer med indendørs trappegang og behøver hjælp til at gå i bad og kan eventuelt have brug for minimal hjælp til påklædning (stikord, let støtte ved behov).
7 Severely Frail – Completely dependent for personal care, from whatever cause (physical or cognitive). Even so, they seem stable and not at high risk of dying (within ~6 months).	7 **Living with Severe Frailty** – Completely dependent for personal care, from whatever cause (physical or cognitive). Even so, they seem stable and not at high risk of dying (within ~6 months).	7: Svært skrøbelig – Fuldstændig afhængige af hjælp til egenomsorg, uanset årsag (fysisk eller kognitiv). Alligevel virker de stabile og ikke i høj risiko for at dø (indenfor ca. 6 måneder).	7: **Lever med svær skrøbelighed** – Fuldstændig afhængige af hjælp til egenomsorg, uanset årsag (fysisk eller kognitiv). Alligevel virker de stabile og ikke i høj risiko for at dø inden for ca. 6 måneder.

8 Very Severely Frail – Completely dependent, approaching end of life. Typically, they could not recover even from a minor illness.	8 **Living with Very Severe Frailty** – Completely dependent **for personal care and** approaching end of life. Typically, they could not recover even from a minor illness.	8: Meget svært skrøbelig – Fuldstændig afhængige og nærmer sig livets afslutning. Typisk vil de ikke engang komme sig efter let sygdom.	8:– Fuldstændig afhængige **af hjælp til personlig pleje** og nærmer sig livets afslutning. Typisk vil de ikke engang komme sig efter let sygdom.
9 Terminally Ill – Approaching the end of life. This category applies to people with a life expectancy <6 months, who are not otherwise evidently frail.	9 Terminally Ill – Approaching the end of life. This category applies to people with a life expectancy <6 months, who are not otherwise **living with severe frailty. (Many terminally ill people can still exercise until very close to death.)**	9: Terminalt syg – Mennesker som nærmer sig livets afslutning, men ikke fremstår tydeligt skrøbelige i øvrigt. Denne kategori gælder mennesker med en forventet levetid på mindre 6 måneder.	9: Terminalt syg – Mennesker **der** nærmer sig livets afslutning. Denne kategori gælder mennesker med en forventet levetid på mindre end 6 måneder, **som ikke lever med svær skrøbelighed i øvrigt (Mange terminalt syge mennesker kan stadig motionere helt indtil livets afslutning).**
Scoring frailty in people with dementia	Scoring frailty in people with dementia	Bedømmelse af skrøbelighed hos mennesker med demens	Bedømmelse af skrøbelighed hos mennesker med demens
The degree of frailty corresponds to the degree of dementia. Common symptoms in mild dementia include forgetting the details of a recent event, though still remembering the event itself, repeating the same question/story and social withdrawal.	The degree of frailty **generally** corresponds to the degree of dementia. Common symptoms in mild dementia include forgetting the details of a recent event, though still remembering the event itself, repeating the same question/story and social withdrawal.	Mennesker med demens er skrøbelige (scorer altid minimum 5) og graden af skrøbelighed svarer til graden af demens. Typiske symptomer ved mild demens er at glemme detaljer om en nylig begivenhed, selvom man kan huske selve begivenheden, og at gentage det samme spørgsmål/historie og social tilbagetrækning.	Mennesker med demens er **oftest** skrøbelige (scorer **oftest** minimum 5) og graden af skrøbelighed svarer **som regel** til graden af demens. Typiske symptomer ved mild demens er at glemme detaljer om en nylig begivenhed, selvom man kan huske selve begivenheden og at gentage det samme spørgsmål/historie og social tilbagetrækning.
In moderate dementia, recent memory is very impaired, even though they can remember their past life events well. They can do personal care with prompting.	In moderate dementia, recent memory is very impaired, even though they can remember their past life events well. They can do personal care with prompting.	Ved moderat demens er hukommelsen for nylige begivenheder svært nedsat, selvom man tilsyneladende kan huske gamle minder tydeligt. Man kan udføre personlig pleje med vejledning.	Ved moderat demens er hukommelsen for nylige begivenheder svært nedsat, selvom man kan huske gamle minder tydeligt. Man kan udføre personlig pleje med vejledning.
In severe dementia they cannot do personal care without help.	In severe dementia they cannot do personal care without help.	Ved svær demens kan man ikke udføre personlig pleje uden hjælp.	Ved svær demens kan man ikke udføre personlig pleje uden hjælp.
	**In very severe dementia they are often bedfast. Many are virtually mute.**		**Ved meget svær demens er man ofte sengeliggende. Mange er nærmest ophørt med at tale.**
*Canadian Study on Health & Aging. Revised 2008	**Clinical Frailty Scale © 2005-2020 Rockwood, Version 2.0 (EN).** All rights reserved. **For permission:** www.geriatricmedicineresearch.ca Rockwood K et al. A global clinical measure of fitness and frailty in elderly people. CMAJ 2005:173;489-495.	* 1. Canadian Study on Health & Aging, Revised 2008. Danish Version I, 2020, translated by Anders Fournaise and Søren Kabell Nissen, University of Southern Denmark.	**Clinical Frailty Scale © 2005-2020 Rockwood, Version 2.0 (EN).** All rights reserved. **For permission:** www.geriatricmedicineresearch.ca Rockwood K et al. A global clinical measure of fitness and frailty in elderly people. CMAJ 2005:173;489-495. **Danish Version 2.0**, 2020, translated by Anders Fournaise and Søren Kabell Nissen, University of Southern Denmark.
